# Supplementary material for: A Microenvironment-Related Nine-Gene Signature May Predict Survival in Mycosis Fungoides Patients at Diagnosis
Source: Cells. 2023 Jul 27;12(15):1944. doi: 10.3390/cells12151944 (PMC10417031; doi:10.3390/cells12151944)
Supplement: Supplementary file 1 [file cells-12-01944-s001.zip › Supplementary File FINAL.pdf]

Supplementary File

Table S1. Genes deregulated in low-risk vs high-risk discovery set

Genes	Log2 fold change	P-value
CCL13	2.7	0.00
CCL18	3.2	0.00
CCL17	3.3	0.00
CCL26	3.3	0.00
EOMES	2.1	0.00
TAL1	2.1	0.00
KLRG1	1.6	0.00
RRAD	1.9	0.00
CD209	1.2	0.00
IL1RL1	1.4	0.00
CXCR3	1.5	0.00
TREM2	2.1	0.00
TPSAB1	1.2	0.00
CD180	1.9	0.00
MME	1.9	0.00
CCR2	1.3	0.00
ITGAM	1.1	0.00
GZMK	1.6	0.00
EGR2	0.9	0.00
IL2RA	-1.3	0.00
CR1	1.5	0.00
BTK	0.8	0.00
CTSG	0.9	0.00
GZMH	1.5	0.00
IL22RA2	1.5	0.00
COLEC12	0.6	0.01
CDH5	0.7	0.01
STAT4	1.0	0.01
CD8B	1.2	0.01
CD8A	1.2	0.01
BID	1.0	0.01
IFNAR1	0.6	0.01
NFATC4	0.6	0.01
PDGFC	0.7	0.01
ST6GAL1	0.7	0.01
CLEC4A	0.8	0.01
CFI	0.9	0.01

F13A1	1.0	0.01
PPARG	1.1	0.01
CSF2	1.6	0.01
IL13	1.8	0.01
C3AR1	0.9	0.01

Table S2. Genes deregulated in low-risk vs high-risk validation set

**Genes**	**Log2 fold change**	**P-value**
CCL13	3.16	0.00
CD209	2.37	0.00
IL23A	-1.79	0.00
CD63	0.81	0.00
CCL26	4.10	0.00
CSF2	3.77	0.00
CD59	1.05	0.00
PPARG	2.30	0.00
CLEC4A	1.57	0.00
CD36	1.93	0.00
NRP1	0.91	0.00
F13A1	1.53	0.00
BCL10	0.44	0.00
CD1A	2.40	0.00
LAMP2	0.71	0.00
TNFSF12	0.70	0.00
LTF	3.30	0.00
IL1R2	1.56	0.00
CD207	2.61	0.00
TBK1	0.51	0.00
IL13RA1	0.63	0.00
CXCL12	2.04	0.00
JAM3	0.89	0.00
VEGFA	1.22	0.00
CMA1	1.95	0.00
SAA1	2.71	0.00
TARP	-2.20	0.00
TNFRSF11A	1.96	0.00
CARD9	1.39	0.00
CD276	0.81	0.00
PRKCE	1.27	0.00
CTSG	1.73	0.00
BCL6	0.82	0.00

NOD1	0.73	0.00
CCL17	2.96	0.00
CD27	-1.69	0.00
CCL18	2.13	0.00
CD33	0.97	0.00
CD244	2.35	0.00
CDH5	1.08	0.00
CD247	-1.58	0.00
TIRAP	0.59	0.00
DEFB1	1.43	0.00
MRC1	1.23	0.00
IFIT2	1.54	0.00
FUT7	1.56	0.00
C1R	0.99	0.00
IL1RAP	0.95	0.00
COL3A1	1.49	0.00
IL1R1	0.79	0.00
TXNIP	0.75	0.00
LRP1	1.07	0.00
MFGE8	0.82	0.00
NCF4	0.86	0.00
RRAD	1.15	0.00
MAF	0.86	0.00
PRAME	-2.14	0.00
TPSAB1	1.38	0.00
TANK	0.50	0.00
HLA-DRB3	0.87	0.00
CCL14	1.17	0.00
ITGB3	1.24	0.00
ETS1	-0.88	0.00
MICA	0.65	0.00
KLRG1	-1.74	0.00
CXCL1	1.90	0.00
PDGFRB	0.95	0.00
CFD	1.82	0.00
HLA-DMA	0.64	0.00
FCER1A	1.84	0.00
S100B	1.54	0.00
FOXP3	1.53	0.00
IGF1R	0.75	0.00
APP	0.71	0.00
LGALS3	1.03	0.00
CXCR6	1.60	0.00
CD70	-1.59	0.00

MAP4K2	-0.59	0.00
ITGA1	1.06	0.00
ANXA1	0.77	0.00
TNFSF10	0.66	0.00
SERPING1	0.87	0.00
TCF7	-1.41	0.00
SBNO2	0.58	0.00
CD1E	1.53	0.00
CD1C	1.40	0.00
IL15RA	0.59	0.00
PLAU	0.74	0.00
BST1	0.85	0.00
KIT	1.42	0.00
IL1RL2	1.26	0.00
AXL	0.83	0.00
CSF1	0.91	0.00
BIRC5	-1.03	0.00
PDCD1LG2	1.01	0.00
ITGAM	0.92	0.00
GZMB	1.92	0.00
CDKN1A	0.71	0.00
CD3EAP	-0.52	0.00
CD1B	1.81	0.00
BTLA	-1.26	0.00
BCL2L1	0.38	0.00
CCL22	1.28	0.00
ITGB4	1.09	0.00
NFATC1	-0.57	0.00
PECAM1	0.56	0.00
KLRD1	-1.16	0.00
IL11RA	0.59	0.00
TRAF6	0.45	0.00
IL6ST	0.40	0.01
NOD2	0.77	0.01
FAS	0.72	0.01
THBD	0.66	0.01
PSEN2	0.61	0.01
FCER1G	0.82	0.01
IL34	0.87	0.01
LAMP1	0.44	0.01
NFATC4	0.64	0.01
EPCAM	-1.39	0.01
CD34	0.87	0.01
C1S	0.79	0.01

LAIR2	-1.00	0.01
NFATC3	-0.50	0.01
CXCR2	0.85	0.01
CD9	0.92	0.01
CEBPB	0.70	0.01
FCGR2B	0.94	0.01
JAK2	0.44	0.01
LILRA1	-1.00	0.01
LAMP3	0.82	0.01
TLR6	0.50	0.01
SIGLEC1	0.81	0.01
LCK	-0.74	0.01
CD5	-1.05	0.01
SIGIRR	-0.69	0.01
ENG	0.58	0.01
CD274	1.05	0.01
SERPINB2	1.43	0.01
RORC	1.63	0.01
LTA	-1.11	0.01
MAP3K5	0.43	0.01
ITGA2	0.82	0.01
IL12RB2	1.49	0.01
CTSW	1.64	0.01
C2	0.63	0.01
CD40	0.52	0.01
ITGA5	0.45	0.01
THY1	0.58	0.01
TAPBP	0.42	0.01
CCL2	0.75	0.01
CD28	-1.07	0.01
CD44	0.48	0.01
CSF1R	0.65	0.01
MME	1.06	0.01
TLR5	0.78	0.01
STAT4	1.01	0.01
UBC	0.39	0.01
CEACAM6	1.26	0.01
CREB5	0.77	0.01
IL16	-0.59	0.01
ICAM2	-0.50	0.01
PRKCD	0.35	0.01
CD81	0.42	0.01
NLRP3	0.70	0.01
FCGR2A	0.76	0.01

CYFIP2	-0.61	0.01
CD80	1.09	0.01
IL2RG	-0.74	0.01
IL1RN	0.97	0.01
LTBR	0.56	0.01
CCL1	2.22	0.01
IFIH1	0.50	0.01
